# The Role Silver Nanoparticles Plays in Silver-Based Double-Perovskite Nanocrystals

**DOI:** 10.1021/acs.chemmater.0c04536

**Published:** 2021-03-17

**Authors:** Shai Levy, Sasha Khalfin, Nicholas G. Pavlopoulos, Yaron Kauffmann, Galit Atiya, Saar Shaek, Shaked Dror, Reut Shechter, Yehonadav Bekenstein

**Affiliations:** † Department of Materials Science and Engineering, Technion-Israel Institute of Technology, Haifa 32000, Israel; ‡ Schulich Faculty of Chemistry, Technion- Israel Institute of Technology, Haifa 32000, Israel; § Research and Exploratory Development Department, The Johns Hopkins University Applied Physics Laboratory, 11100 Johns Hopkins Road, Laurel, Maryland 20723, United States; ∥ The Solid-State Institute, Technion-Israel Institute of Technology, 32000 Haifa, Israel

## Abstract

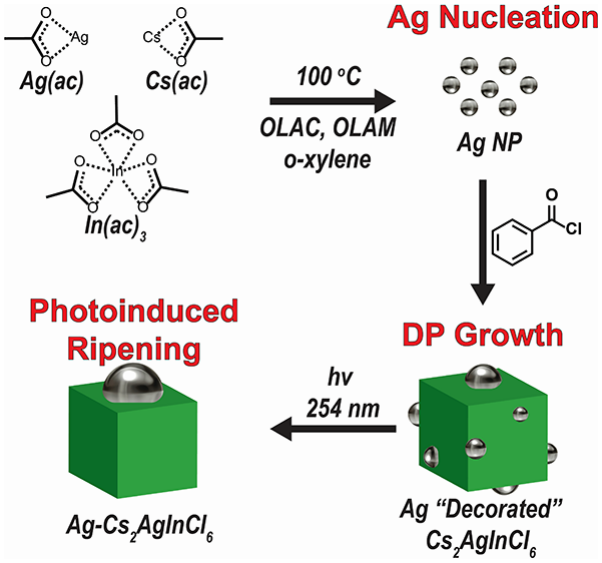

Lead-free double perovskites are studied as an optional replacement to lead halide perovskites in optoelectronic applications. Recently, double-perovskite materials in which two divalent lead cations are replaced with an Ag^+^ and a trivalent cation have been demonstrated. The presence of a reactive silver cation and observations of metallic silver nanodecorations raised concerns regarding the stability and applicability of these materials. To better understand the nucleation and crystal growth of lead-free double perovskites, we explore the origin and role that metallic silver nanoparticles (NPs) play in the Ag-based Pb-free double-perovskite nanocrystal (NC) systems such as Cs_2_AgInCl_6_, Cs_2_AgSbCl_6_, Cs_2_AgBiCl_6_, and Cs_2_AgBiBr_6_. With major focus on Cs_2_AgInCl_6_ NCs, we show evidence supporting growth of the NCs through heterogeneous nucleation on preexisting metallic silver seeds. The silver seeds nucleate prior to injection of halide through reduction of the Ag^+^ ion by the aminic ligand. The presence of preexisting silver NPs is supported by a localized surface plasmon resonance (LSPR). The injection of halide precursor into the reaction mixture step initiates a fast nucleation and growth of the perovskite NC on the silver seed. The change in the dielectric medium at the interface of the silver NP results in a quantifiable red shift of the LSPR peak. In addition, we demonstrate charge transfer from the perovskite to the silver NP through photoinduced electrochemical Ostwald ripening of the silver NPs via UV irradiation. The ripened perovskite–metal hybrid nanocrystal exhibits modified optical properties in the form of quenched emission and enhanced plasmonic absorption. Future development of Ag-based double-perovskite NC applications depends on the ability to control Ag^+^ reduction at all synthetic stages. This understanding is critical for delivering stability and functionality for silver-based lead-free perovskite nanocrystals.

## Introduction

**Figure 1:**
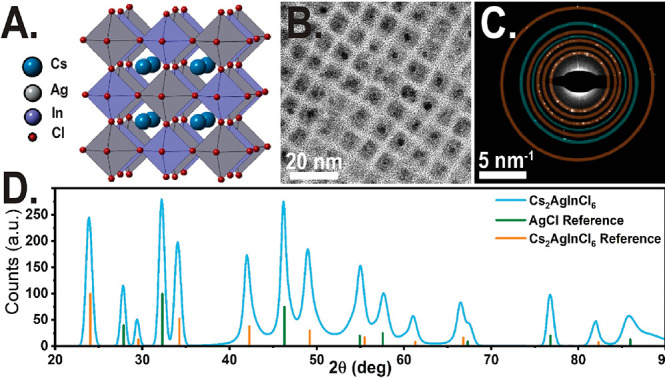
(A) Cs_2_AgInCl_6_ double-perovskite crystallographic structure. (B) Low-resolution TEM of rectangular Cs_2_AgInCl_6_ NCs with high-contrast spherical NPs decorations. (C) SAED of Cs_2_AgInCl_6_ NCs presenting a polycrystalline ring pattern. SAED reveals two phases assigned to Cs_2_AgInCl_6_ and metallic Ag or AgCl. (D) XRD pattern of Cs_2_AgInCl_6_NCs; diffraction shows two distinct phases assigned for Cs_2_AgInCl_6_ (bulk references ICDD no. 01-085-7533) and AgCl (ICDD no. 1-1013).

The study of colloidal cesium lead halide perovskite (LHP) nanocrystals has grown rapidly in recent years due to their attractive optoelectronic properties. LHP NCs have been employed in various applications, such as solar cells, light-emitting diodes, and photodetectors.^1,2^ However, the use of lead remains a major setback due to its toxicity^3,4^ and water solubility.^5^ This has accelerated the study of alternative stable and environmentally sustainable metal halide perovskite NC systems. One optional replacement of lead is using a combination of a monovalent and a trivalent cations to form double perovskites (DP) with a stoichiometry of *A*_2_*M*^+^*M*^+3^*X*_6_(6−9) (as seen in Figure 1A). Initially, research was centered on Cs_2_AgBiBr_6_,^10−12^ which is an indirect band-gap semiconductor exhibiting long excited state lifetimes.^13^ Alternative cations have also been explored, leading to recently reported direct band-gap Cs_2_AgTlBr_6_^14^ and Cs_2_AgInCl_6_^15,16^ DP semiconductors. In the Cs_2_AgInCl_6_ system, efforts were made in order to increase the photoluminescence quantum yield for light-emitting applications.^16,17^ This research includes alloying with sodium ions and doping with various elements such as Bi,^18−20^ Mn,^21^ and Yb^20,22^ ions. The presence of a silver component in a halide perovskite crystal matrix, which is infamously known for its low melting temperatures, facile anion exchange,^23^ and degradation tendencies, has raised material stability concerns. Here, we investigate a common inhomogeneity in the silver-containing DP NCs. These inhomogeneities present themselves as small, seemingly spherical, and high-contrast NPs under transmission electron microscopy (TEM) characterization.^10,14^ Past structural and elemental analyses of these NPs, in both the silver–indium and the silver–bismuth systems, have identified these as metallic silver NPs. However, the origins of these NPs are a subject of an ongoing scientific debate. While studying the silver–indium system, Locardi et al. suggested that reduction of Ag^+^ by the electron beam during TEM characterization is the origin of the metallic silver NPs.^21^ For the silver–bismuth system, the origin of Ag^0^ NPs was assigned to degradation of Cs_2_AgBiBr_6_ NCs,^10^ as supported by the size increase of Ag^0^ NPs decorating the Cs_2_AgBiBr_6_ NCs with increased reaction time. It is worth noting that similar inhomogeneities are observed also in other perovskite NCs. For example, in the more explored lead halide perovskite nanocrystals, high-contrast NPs are also observed. Those NPs are identified as metallic lead NPs, which further emphasizes the chemical analogy between LHP and lead-free DP colloidal systems. While many researchers assigned these to post synthesis degradation,^24,25^ an alternative explanation claiming lead NPs act as nucleation seeds for LHP nucleation was presented by Udayabhaskararao et al.^26^ This claim was supported by the presence of the metallic lead NPs prior to addition of Cs to the reaction mixture and, therefore, predating the formation of LHP nanocrystals. Intrigued by this scientific debate, we set out to explore empirical evidence in order to determine the role of silver NPs in lead-free perovskites.

Here, we synthesized Ag-based Pb-free DP NCs using a modified colloidal synthesis protocol and confirmed the chemical identity of the high-contrast decorations to be metallic Ag NPs. With the main focus on Cs_2_AgInCl_6_ NCs, TEM and optical spectroscopy were used to establish the existence of silver NPs prior to the growth of DP NCs. We identified a localized surface plasmon response (LSPR) of Ag NPs and its subsequent red shifting once the DP perovskite NCs grow. These results are in agreement with an increase in the dielectric medium from growth of a halide perovskite on the surfaces of preexisting metallic NPs. In addition, in Cs_2_AgInCl_6_ NCs, TEM characterization of the resulting perovskite NCs suggests a correlation between the size and the position of the silver NPs that could be explained with a heterogeneous nucleation process. Lastly, UV light-induced Ostwald ripening of the metallic NPs resulted in recovery of the LSPR. Photoinduced electrochemical ripening of metallic NPs in DP NCs is important for understanding variations in the optical properties and degradation upon excitation; to the best of our knowledge, this phenomenon has not been reported to date for DP NC systems.

## Experimental Methods

### Materials

Antimony acetate (99.99%, Aldrich), benzoyl bromide (99.9%, Aldrich), benzoyl chloride (99.9%, Alfa Aesar), bismuth acetate (99.99%, Aldrich), cesium acetate (99.9%, Aldrich), indium(III) acetate (99.99%, Aldrich), oleic acid (90%, Aldrich), oleylamine (70%, Aldrich), silver acetate (99.99%, Aldrich), *o*-xylene anhydrous (97%, Aldrich), and hexane (97%, Aldrich) were used as purchased without further purification.

### Synthesis of Cs_2_AgInCl_6_ Nanocrystals

In a typical synthesis, 25 mg (0.125 mmol) of cesium acetate, 40 mg (0.25 mmol) of silver acetate, and 80 mg (0.25 mmol) of indium(III) acetate were placed into a 20 mL glass vial with a magnetic stirring bar. *o*-Xylene (5 mL), oleic acid (1.25 mL), and oleylamine (0.375 mL) were added, and the vial was heated to 100 °C for the desired amount of time. Benzoyl chloride (0.200 mL, 1.5 mmol) was injected quickly. Then the vials were taken out of the oil bath and left to cool to room temperature or quenched in a water bath. For measurements, the nanocrystal reaction mixture was precipitated by centrifugation at 12 000 rpm for 10 min. The solution was discarded, and the resulting pellet was redispersed in hexane.

### UV–vis Absorption, PL, and Excitation Measurements (PLE)

For optical measurements, 200 μL of the sample solution was injected to a 96-well microplate or 5 mL of the sample solution in a Take-3 quartz cuvette and measured in a Synergy H1 hybrid multimode reader. The samples were irradiated using a xenon lamp (Xe900).

### Transmission Electron Microscopy (TEM) Characterization

One drop of a dilute nanocrystal solution in hexane (1:20 dilution) was cast onto a TEM grid (carbon film only on 300-mesh copper grid or ultrathin carbon film on a holey carbon support film, 400-mesh copper grid). The samples were observed in TEM mode with a Thermo Fisher/FEI Tecnai G^2^ T20 S-Twin LaB_6_ TEM operated at 200 keV with a 1K × 1K Gatan 694 slow scan CCD. High-resolution imaging, diffraction patterns acquisition, and chemical mapping were done in a Thermo Fisher/FEI Titan-Themis double-Cs-corrected HR-S/TEM operated at 200 kV and equipped with a Ceta2 4K × 4K camera (for TEM mode) and Bruker Dual-X EDX detectors for STEM-EDX chemical mapping. The high-resolution STEM micrographs were acquired using a high-angle annular dark-field (HAADF) STEM detector with a collection angle range of 93–200° mrad and beam convergence of 21° mrad. Tilts of 0° (base state), 44°, and 50° were performed with a camera length of 115 mm. STEM-EDX measurements were acquired and analyzed using the Thermo Fisher Velox software.

### X-ray Diffraction

The nanocrystal solution in hexane was drop cast onto a glass substrate (rectangular microslides, 76 × 26 [mm]), and the X-ray beam was focused on the resulting film. Measurements were taken using a Rigaku Smart-Lab 9 kW high-resolution X-ray diffractometer equipped with a rotating anode X-ray source. We used the “Glancing mode” (grazing angle) method (2-theta), which is suitable for measuring thin films, with a 1.54 Å (Cu Kα) wavelength. The X-ray source was fixed on ω = 0.4°, and the detector was moved in the range of 2θ = 20–90°.

### X-ray Photoelectron Spectroscopy (XPS) Characterization

A few drops of the nanocrystal solution in hexane were cast onto a clean gold substrate. X-ray photoelectron spectroscopy (XPS) measurements were performed in an analysis chamber (UHV – 210–10 Torr during analysis) using a Versaprobe III-PHI Instrument (PHI, USA). The sample was irradiated with a focused Al Kα monochromated X-ray source (1486.6 eV) using an X-ray beam size diameter of 200 μm, 25 W, and 15 kV. The outcoming photoelectrons are directed to a spherical capacitor analyzer (SCA). Sample charging was compensated by dual-beam charge neutralization based on a combination of a traditional electron flood gun and a low-energy argon-ion beam. Survey spectra are presented as a plot of the number of photoelectrons (units of counts/s) measured as a function of the binding energy (units of eV). The survey spectra were collected with a pass energy of 140 eV and a step size of 0.5 eV. The core level binding energies of the different peaks were normalized by setting the binding energy for the C 1s at 284.8 eV.

### UV–vis Irradiation Photocemically Activated Ostwald Ripening Experiment

The same procedure for synthesis of Cs_2_AgInCl_6_ nanocrystals was conducted but under dark conditions. Then the product hexane solution of the reaction after centrifugation was placed in a quartz cuvette and irradiated for the desired amount of time with LED light sources or using an UV lamp. The samples were irradiated for 30 min by 660, 445, and 330 nm 100 mW LED light sources and a 255 nm 5 mW UV lamp. Then TEM grids were casted in the dark from the irradiated solution for TEM characterization. The dark synthesis product solution and the irradiated product solution were measured by UV–vis absorption and PL for optical characterization.

## Results and Discussion

### Ag-Based DP Nanocrystals Characterization

**Figure 2:**
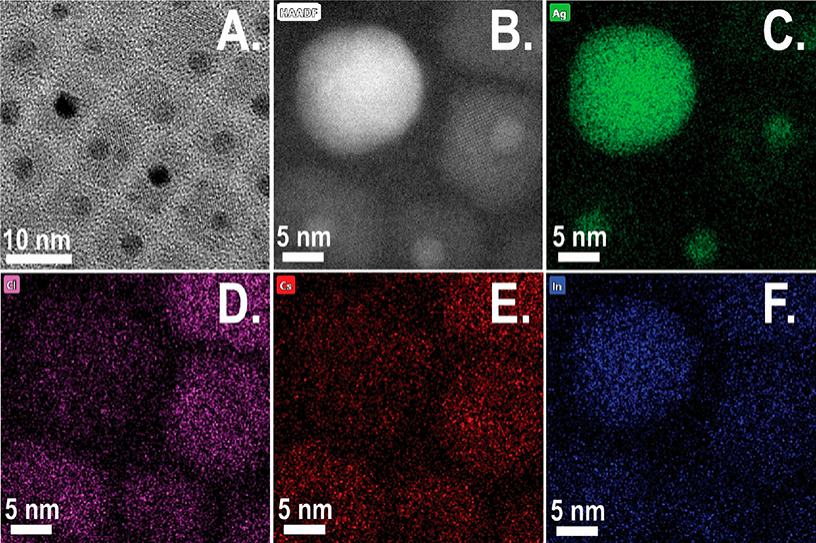
(A) High-resolution TEM of Cs_2_AgInCl_6_ NCs. (B) HAADF-STEM image of Cs_2_AgInCl_6_ NCs with spherical NP decorations and large spherical NP byproducts. (C, d, e, and F) EDX elemental mapping of B image for silver, chlorine, cesium, and indium, respectively. Identity of both the decorations and the byproduct NP is assigned to metallic silver.

The synthesis of Cs_2_AgInCl_6_ NCs was performed using a colloidal hot-injection approach in which metal acetate (ac) precursors (i.e., Ag(ac), In(ac)_3_, and Cs(ac)) were first dissolved in *o*-xylene together with organic ligand surfactants (oleylamine (OLAM) and oleic acid (OLAC). The precursors solution was heated for the desired complexation time until benzoyl chloride was swiftly injected to induce nucleation and growth of Cs_2_AgInCl_6_ NCs. After the desired reaction time, the reaction solution was quenched in an ice–water bath, cleaned, and redispersed in hexene. Similar syntheses were performed in order to synthesize Cs_2_AgBiCl_6_, Cs_2_AgBiBr_6_, and Cs_2_AgSbCl_6_ NCs (see Supporting Information). TEM characterization of the Cs_2_AgInCl_6_ NCs shows 5–10 nm rectangular-shaped NCs (Figure 1B). As seen in the TEM images, the cubic-shaped Cs_2_AgInCl_6_ NCs are decorated by high-contrast spherical NPs. To verify the crystal structure, XRD study of the end products was conducted and confirmed the presence of DP Cs_2_AgInCl_6_ phase (ICDD no. 01-085-7533) as well as a silver chloride byproduct (ICDD no. 1-1013) (as shown in Figure 1D). Selected area electron diffraction (SAED), seen in Figure 1C, resulted in a polycrystalline ring pattern, indicating two phases. These phases were determined to be representative of Cs_2_AgInCl_6_ in addition to either metallic silver or AgCl. A fully analyzed image of the selected area for the SAED is shown in the Supporting Information (Figure S1). To further probe the identity of the higher contrast spherical decorations, we conducted high-resolution (HR) TEM and high-angle annular dark-field scanning TEM (HAADF-STEM) as shown in Figure 2A and 2B. Furthermore, we conducted HR energy-dispersive X-ray spectroscopy (EDX) elemental mapping (shown in Figure 2C–F). The EDX elemental mapping and fitting for the cuboid-shaped nanocrystals resulted in a composition of 21.08 ± 2.87% Cs, 9.21 ± 1.35% Ag, 9.68 ± 1.41% In, and 60.03 ± 5.00% Cl. This result is in strong agreement with previous reports for Cs_2_AgInCl_6_.^16,21^ High-resolution elemental mapping for several of the high-contrast morphologically distinct spherical NPs showed a higher Ag composition, as high as 92.52 ± 20.16% Ag, indicating the spherical NPs are indeed metallic silver. This agrees with the low-resolution EDX mapping by Dahl et al.^16^ for the Cs_2_AgInCl_6_ system and Bekenstein et al.^10^ for the Cs_2_AgBiBr_6_ system.

### Two Types of Silver NP Decorations

**Figure 3:**
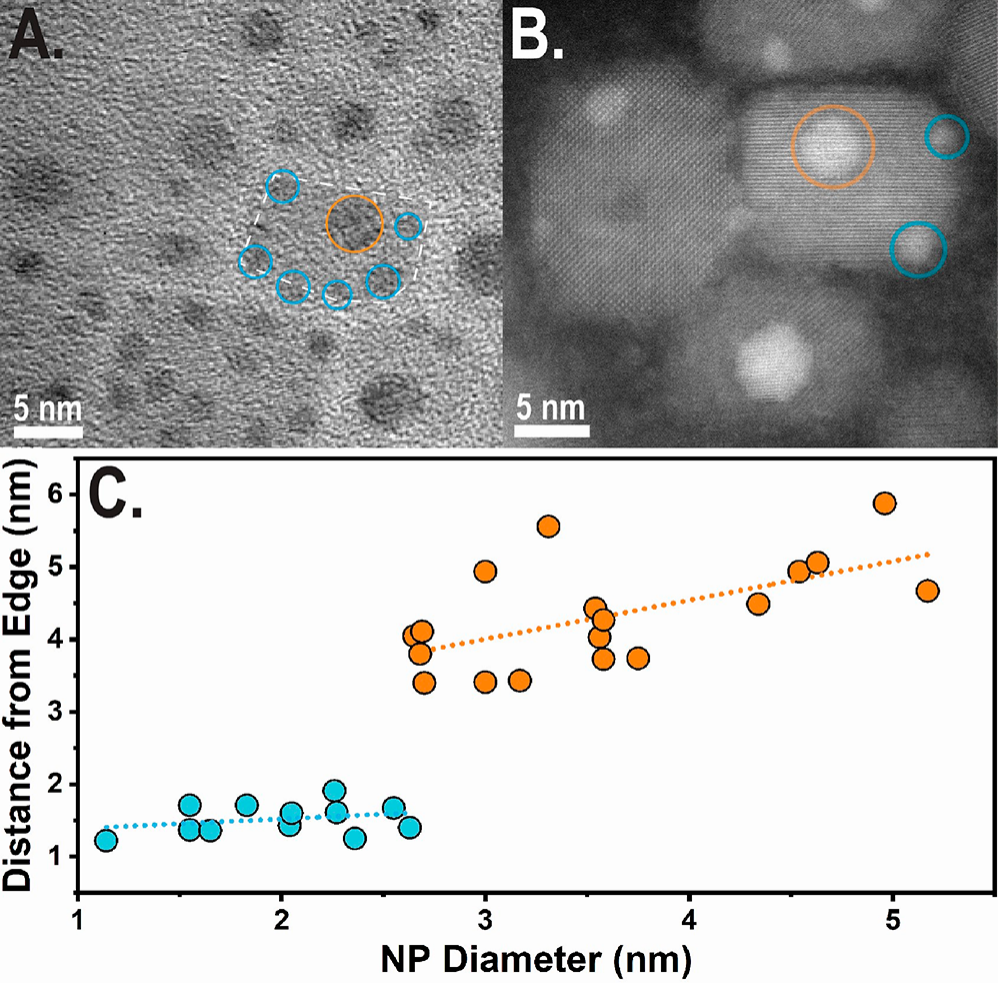
(A) High-resolution TEM and (B) high-resolution HAADF-STEM of Cs_2_AgInCl_6_ NCs exhibiting multiple and various sizes of silver NPs decorations per nanocube. Smaller silver NPs are located near the edges and corners of the nanocube, while main central silver NP is larger. (C) Plot demonstrating correlation of silver NPs diameter vs distance from the edge of the Cs_2_AgInCl_6_ NC. Two distinct populations are portrayed in the central primary NP population (orange) further from the edges and the small secondary decorations NP (blue) closer to the edges.

In order to determine the origin and role of the spherical metallic silver NPs in the Cs_2_AgInCl_6_ NCs colloidal system, we surveyed several HR-TEM images of the Cs_2_AgInCl_6_ NCs with decorations from different synthetic batches. In most of the Cs_2_AgInCl_6_ NCs, multiple Ag NPs decorations can be found on the same rectangular Cs_2_AgInCl_6_ NC (Figure 3A and 3B). Upon closer inspection, two different populations of Ag NPs can be differentiated with a distinct size difference: a large centralized NP (primary population) along with smaller surrounding silver decorations (secondary population). The primary NP is typically located near the center of the cuboid in the TEM projected image (not necessarily a core–shell structure). The smaller decorations tend to mainly be located at the edges and corners of the Cs_2_AgInCl_6_ NCs. There is a clear correlation between the position of the silver decoration relative to the NC edge and the decoration’s diameter, as seen in Figure 3C, suggesting two distinct modes of growth for these different Ag NPs. Closer examination of the silver decorations at the edges and corners of the particle (secondary population) reveal cases where the NPs were partially located outside of the Cs_2_AgInCl_6_ NCs projection. This indicates that the silver NPs secondary population may reside on surfaces of the Cs_2_AgInCl_6_ NC and not necessarily embedded inside them. We recall that the corners and edges of the Cs_2_AgInCl_6_ NC have low effective concentrations of surfactant ligands, making them more favorable for Ag NPs adsorption. Similarly, a low ligand density was associated with selective deposition of Au NPs in CdSe@CdS tetrapods, nanorods,^26−28^ and CsPbBr_3_;^29^ secondary silver NPs are being adsorbed or reduced onto the edges and surfaces post growth of the perovskite crystal or are the result of degradation. Now, we discuss the large centralized decoration (primary population). In order to determine whether the NPs are embedded in a core–shell structure or on the surfaces of the crystals, we conducted an aberration-corrected high-resolution TEM tilting experiment. By changing the angle of the TEM grid we discovered that the central large Ag decorations are located on the surface of the Cs_2_AgInCl_6_ NCs as well (Figure S3). This is evident by the large central decoration located partially outside the projected edge of the Cs_2_AgInCl_6_ NCs when the tilting angle increases (images are shown in Figure S3).

### Silver NPs Seed Mediated Nucleation of Ag-Based DP Nanocrystals

**Figure 4:**
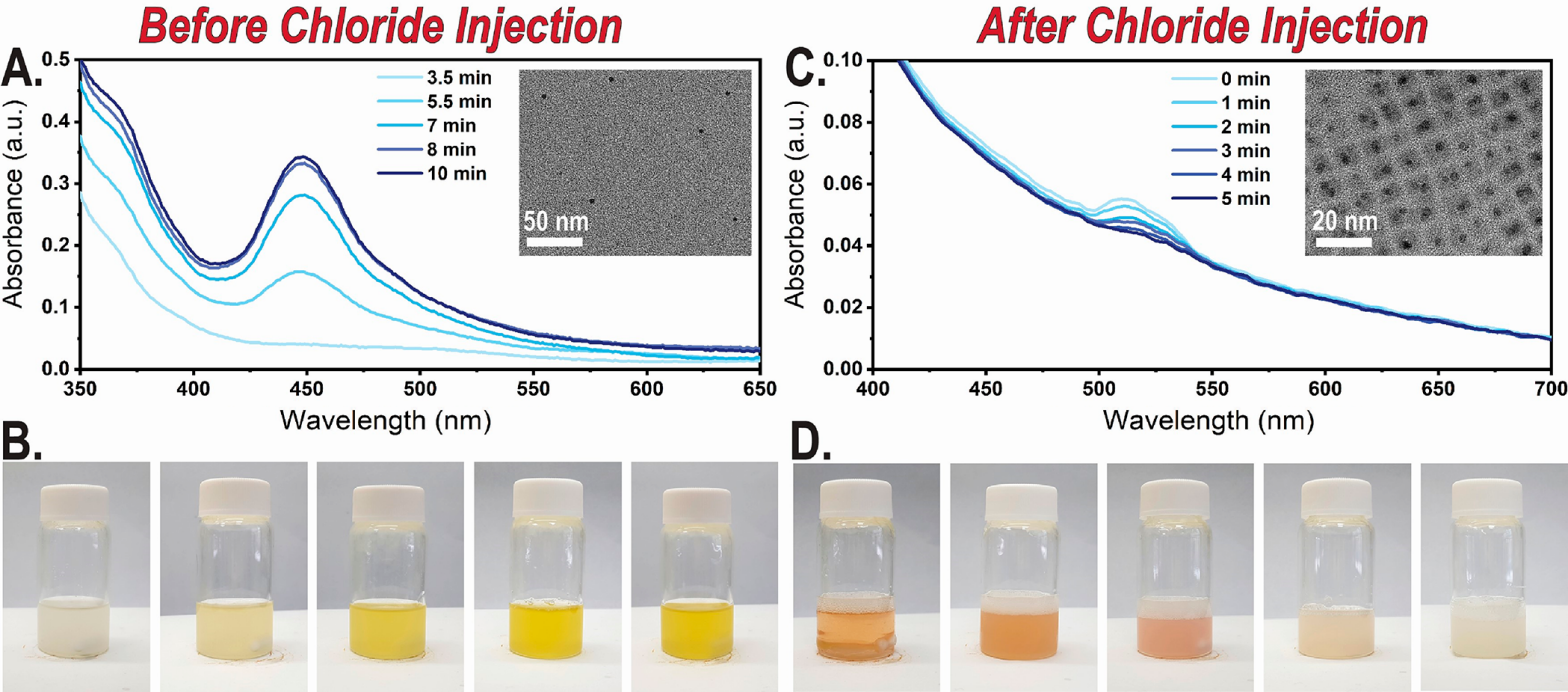
(A) Absorbance spectrum of 45 °C reaction mixture at different times at the complexation stage. Formation of metallic silver NPs is indicated by the increase in the localized surface plasmon resonance (LSPR) peak. (Inset) Low-resolution TEM image of metallic silver NPs formed during complexation time. (B) Images of the reaction solution at different complexation times at 45 °C (from left to right) at 0, 3.5, 5.5, 8, and 10 min. (C) Absorbance spectrum of a diluted reaction mixture at different reaction times at 25 °C after ice bath quenching. LSPR peak is shifted from 450 (as seen in A) to 520 nm, and overall scattering increases. (Inset) Low-resolution TEM image of metallic silver NPs decorating Cs_2_AgInCl_6_ NCs after injection of chloride and reaction. (D) Images of the reaction solution at different reaction times at 45 °C (from left to right) at 0, 2, 4, 7, and 10 s.

In order to understand the stage in the reaction in which the silver NPs form and their role in the perovskite crystal growth, we conducted a series of synthetic experiments. We observed that the overall complexation time has a major effect on the color of the precursor’s solution. Figure 4A and 4B shows the color change of the solution from clear to yellow-orange with increased intensity at longer complexation times. We assign this color change to a silver surface plasmon resonance. Fundamentally, the plasmon appearance indicates formation of metallic silver NPs due to the reduction of Ag–acetate by oleylamine during the complexation stage which is prior to chloride injection and perovskite formation. Formation of metallic silver NPs from Ag^+^ ions in the presence of both aliphatic ligand (oleic acid) and reducing agent (oleylamine) is well known in the scientific literature and is used as one of the main synthetic methods for synthesizing metallic silver colloids.^27−29^ The presence of Ag NPs in the complexation stage was also confirmed by TEM imaging (Figure 4A) along with XRD analysis (Figure S5). XPS analysis on the end product of the reaction identified metallic silver prior to any electron beam exposure (Figure S5). A similar silver LSPR was observed in the silver–bismuth NC precursor solution during complexation (see Supporting Information).

Next, we examined the chloride injection stage of the reaction. In a typical reaction at 100 °C, the yellow-orange color of the solution changes rapidly as a result of the chloride injection to clear and then murky white in a matter of only a few seconds. This rapid reaction is limiting characterization approaches. In order to learn more about the chemical and physical changes during injection, a series of reactions was conducted at a lower temperature (45 °C), thus slowing the kinetics of the reaction significantly. Images and absorbance spectra of these experiments are shown in Figure 4C and 4D. After chloride injection, the plasmon peak shifts from 450 to 520 nm (generally associated with an increase in Ag NP size, or a change in dielectric medium). Surprisingly, size distribution analysis of the Ag NPs in TEM imaging demonstrates a decrease in the average size of the Ag NPs after injection of chloride, from 3.5 ± 0.6 to 2.2 ± 0.4 nm (Figure S6), while the smaller NP size suggests partial consumption of Ag NPs occurring during formation of the Cs_2_AgInCl_6_ NCs and AgCl byproduct. We could not rule out that the consumption of silver NPs is the cause for the plasmon reduced intensity (Figure 4C); however, a short time after injection, many free silver NPs were identified (see Supporting Information). Smaller Ag NPs should also result in a blue shift and not the observed red shift of the plasmon (Figure 4A–C). Indeed, the shifted peak of the plasmon could not be explained based on the size difference of the silver NPs. We hypothesize that the plasmon red shift is a result of the growth of DP NCs on the preexisting silver seeds. A similar plasmonic change occurs in the silver–bismuth NC systems examined during injection of benzoyl halide. However, in these systems the reaction rate is higher and does not allow for absorbance measurements during the plasmonic shift even at 45 °C.

To further understand the shift in the Ag NP plasmon resonance, a fundamental discussion of the physical origin of the light absorption by the metallic silver NPs as a plasmon is warranted. When incident photon frequency is resonant with the collective oscillation of the conduction band electrons in the Ag NP, a characteristic absorbance peak is observed, and it is known as a localized surface plasmon resonance (LSPR). The resonance frequency of this LSPR is strongly dependent on the composition, size, and shape of the NPs but is also depended on the dielectric properties of the surrounding medium.^28,30−32^ The correlation between the maxima of the LSPR and the dielectric constant (or refractive index) of the surrounding medium can be treated within the framework of the Drude model.^33−35^ The surface plasmon peak wavelength (λ) is related to the refractive index of the surrounding medium (*n*) by the following expression
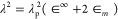
1where λ_p_ is the bulk metal plasmon wavelength, ∈^∞^ is the high-frequency dielectric constant, and ∈_*m*_ (∈_*m*_ = *n*^2^) is the optical dielectric function of the medium. Substituting eq 1 with the LSPR peak wavelength of 450 nm (as seen in Figure 4A), metallic silver’s bulk plasmon wavelength (138 nm^33^), and the optical dielectric function of *o*-xylene to 2.28^36^ results in a high-frequency dielectric constant of 6.07. This result is aligned with the reported value in the literature.^33^ In order to explain the red-shifted LSPR peak at the addition of the chloride source (as seen in Figure 4D), we calculated the expected shift of the LSPR peak due to a change in the dielectric function of the medium as a result of the growth of Cs_2_AgInCl_6_NCs. This is done by substituting eq 1 with the same value for the bulk metal plasmon wavelength using the high-frequency dielectric constant found before and approximating the optical dielectric function of Cs_2_AgInCl_6_ to be (∈_*m*_ ≈ 4), based on the reported dielectric function for Cs_2_BiCuCl_6_,^37^ Cs_2_AgBiCl_6_,^38^ and Cs_2_AgInCl_6_^39^ near 450 nm. This substitution results in a LSPR peak at 517.6 nm. This value is in strong agreement with the observed LSPR in Figure 4D at 520 nm after initiation of perovskite NC growth. This strongly supports heterogeneous nucleation of Cs_2_AgInCl_6_NCs at the surface of the preexisting metallic silver NPs post chloride injection. The model of eq 1 assumes that the Ag sphere is completely embedded in the dielectric medium. However, the presence of ligands modifies the effective dielectric medium for the NPs. The converage of ligands is likely to change during the DP nucleation and growth in the reaction, making the dielectric medium approximation more complicated as mentioned in other LSPR studies.^40^

By the previously presented hypothesis, we now find an explanation for the different populations of Ag NPs present in the end product of the reaction using classical nucleation theory (CNT). In a comparison between the homogeneous and the heterogeneous nucleation processes of Cs_2_AgInCl_6_NCs, many parameters are required. Such parameters include the volume and free surface area of the Cs_2_AgInCl_6_NC and Cs_2_AgInCl_6_ surface energy and the silver NP defect surface energy. However, the favorability of heterogeneous nucleation over homogeneous nucleation is clear due to the reduction in the defect energy (Ag NP surface energy) in the heterogeneous process. Therefore, the favorability for heterogeneous nucleation is proportional to the size of the Ag NP.^41,42^ In such a case, it is feasible to expect that the large (primary) Ag NP on the Cs_2_AgInCl_6_NC is the nucleation seed for DP nucleation, while the small silver NP decorations (secondary) are likely the result of postsynthesis attachment or degradation process. This population of small NPs remains free from perovskite growth since heterogeneous nucleation favors larger Ag NPs. The small free Ag NPs are attached mainly on the edges and corner of the Cs_2_AgInCl_6_NC due to the lower effective ligand concentrations at these locations as described earlier. Another observation that can be explained via heterogeneous nucleation is the unlikely location of the large (primary) silver NPs on the face of the DP NCs where ligand concentration is high, while the much smaller Ag NPs (secondary) are adsorbed almost exclusively on less ligand-passivated locations (corners and edges). This can be explained since growth of DP is favorable on larger silver NPs that serve as seeds for nucleation, thus preexisting the facet and its passivating ligands. The smaller NPs (secondary) that remain unreacted in solution may adsorb to less passivated areas, such as corners and edges. This therefore explanans the size–location correlation of the silver NPs reported in Figure 3A.

### Photochemically Activated Ostwald Ripening of Ag NPs

**Figure 5:**
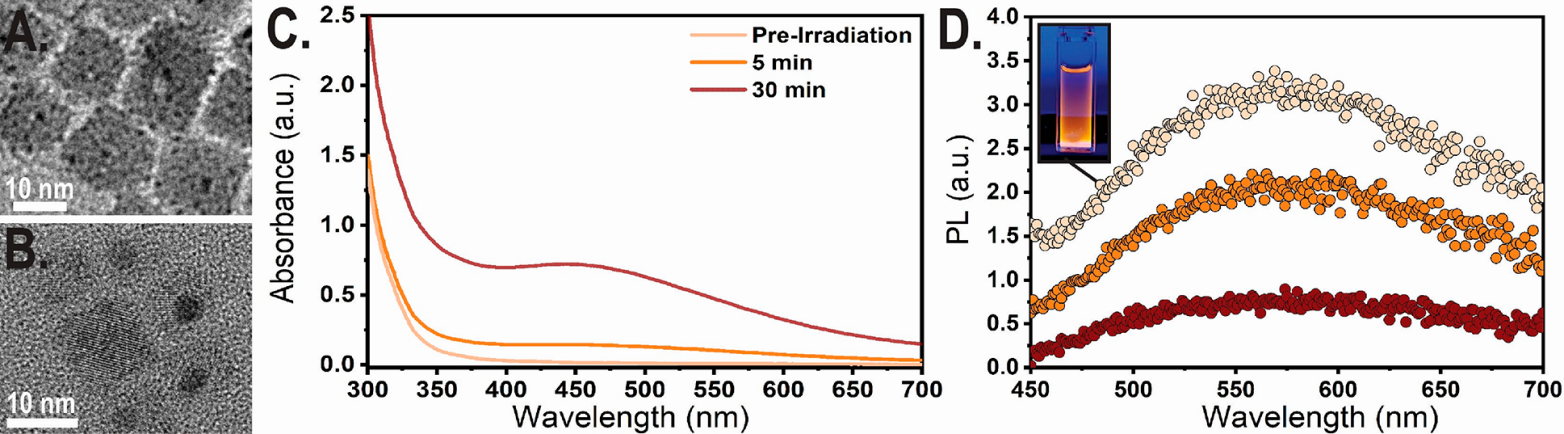
(A) High-resolution TEM micrograph of Cs_2_AgInCl_6_ NCs, made under dark reaction conditions. NCs have multiple small silver NPs decorations per nanocube in different locations. (B) High-resolution TEM micrograph of Cs_2_AgInCl_6_ NCs from the sample in A after UV irradiation (254 nm, 5 mW, 30 min). After irradiation there is mostly one large central silver decoration per nanocube as a result of Ostwald ripening. (C) Optical absorbance measurements of Cs_2_AgInCl_6_ NCs before and after UV irradiation treatment. Absorbance spectrum after irradiation has LSPR with a peak at 450 nm forming. (D) PL spectrum of Cs_2_AgInCl_6_ NCs before and after UV irradiation. PL intensity decreases after irradiation. (Inset) Cs_2_AgInCl_6_NCs solution’s visible fluorescent orange emission during the start of the 254 nm irradiation experiment.

Lastly, we tested the influence of UV–vis light irradiation on the silver-decorated DP, which exhibist similar structural characteristics to other metal–semiconductor hybrid colloidal heterosystems.^43−46^ In order to study the effect of radiation on the metallic silver decorations, we synthesized Cs_2_AgInCl_6_NCs under dark conditions and kept the solution this way until TEM characterization. Part of the product solution was placed in a quartz cuvette and irradiated for 30 min with LED light sources or using an UV lamp (660, 445, and 330 nm 100 mW LED light sources and 255 nm 5 mW UV lamp). The sample that was not exposed to irradiation (seen in Figure 5A) demonstrated smaller than usual silver decorations and a large amount of silver decorations. Samples that were exposed to below band-gap irradiation (660, 445, and 330 nm) did not reveal any differences in their silver decorations population. The sample excited with above band-gap irradiation (254 nm) was the only one in which Cs_2_AgInCl_6_NCs were excited and demonstrated a clear visible orange fluorescent emission during irradiation (seen in Figure 5D). TEM characterization of this sample, as seen in Figure 5B, demonstrated some perovskite NCs with only one large central silver NP decoration in contrast to the other samples in this experiment.

We assign this result to a photochemically activated electrochemical Ostwald ripening of Ag NPs induced by charge separation of the exciton across the metal–semiconductor junction at the Cs_2_AgInCl_6_–Ag interface. In this process the smaller and less thermodynamically stable secondary Ag NPs are electrochemically oxidized to free Ag^+^ ions that are released into solution and subsequently reduced by electrons localized in the larger primary Ag NP_,_ resulting in metallic silver deposition onto an existing silver decoration.^47^ This mechanism allows for the optically activated coarsening of the central silver decoration to a more thermodynamic favorable product. Some of the small silver NPs are consumed in this coarsening process, and the remaining NPs are larger, as seen in the comparison between Figure 5A and 5B.

An additional difference between the excited sample and the other nonexcited samples is the difference in the final color of the solution and the integral intensity of the emission, as seen in Figure 5C. The color change of the Cs_2_AgInCl_6_ NCs solution in hexane from clear to red-orange is presented as a peak in the absorbance spectrum in Figure 5C. This absorbance peak could be associated with LSPR due to the resulting photochemically activated Ostwald ripening of Ag NPs in the 254 nm irradiation. Another optical difference is the damping of the fluorescent emission of the Cs_2_AgInCl_6_ solution as a result of the duration of the 254 nm irradiation. This observation can also be explained by the Ag NPs coarsening. Cs_2_AgInCl_6_NC with a large central silver decoration is more likely to undergo nonradiative recombination and therefore a lower intensity of the emission of the irradiated sample, as presented in Figure 5D. Therefore, the mechanism of photochemically activated Ostwald ripening of Ag NPs could be a major part of the degradation in the optical emission properties in the Cs_2_AgInCl_6_ and could have a negative effect in many applications for the Cs_2_AgInCl_6_ NCs, such as display applications.

## Conclusions

In this report, we show empirical evidence of the role that metallic silver NPs plays as heterogeneous nucleation seeds for Ag-based DP NCs. Formation of silver NPs occurs through reduction of the Ag^+^ by amines in the complexation stage of the NCs synthesis. Subsequent injection of halide results in DP NCs heterogeneous nucleation on the preexisting silver NPs. Moreover, we demonstrate optical-induced Ostwald ripening of Ag NPs during Cs_2_AgInCl_6_ excitation. The ripening is accompanied by changes to the optical properties of the suspension, plasmonic absorption, and quenched emission. Understanding the role of the metallic silver in Ag-based DP opens possibilities for the controlled design of lead-free perovskite–metal hybrid nanocrystals. This may lead to the design of new synthetical approaches in order to allow DP homogeneous nucleation and therefore more intense emission.

## Supporting Information Available

XPS data; additional TEM, SAED, and EDS elemental analysis (PDF)
