# Supplementary material for: The Role Silver Nanoparticles Plays in Silver-Based Double-Perovskite Nanocrystals
Source: Chem Mater. 2021 Mar 17;33(7):2370–7. doi: 10.1021/acs.chemmater.0c04536 (PMC8274473; doi:10.1021/acs.chemmater.0c04536)
Supplement: Supplementary file 1 — cm0c04536_si_001.pdf [file cm0c04536_si_001.pdf]

Supporting Information For:

The Role Silver Nanoparticles play in Silver-Based Double Perovskite Nanocrystals

Shai Levy[1], Sasha Khalfin[1], Nicholas G. Pavlopoulos[2,3], Yaron Kauffmann[1], Galit Atiya[1], Saar Shaek[1], Shaked Dror[1], Reut Shechter[1] and Yehonadav Bekenstein*[1,4]

1 Department of Materials Science and Engineering, Technion-Israel Institute of Technology, Haifa 32000, Israel
2 Schulich Faculty of Chemistry, Technion − Israel Institute of Technology, Haifa 32000, Israel
3 Research and Exploratory Development Department, The Johns Hopkins University Applied Physics Laboratory, 11100 Johns Hopkins Road, Laurel, Maryland 20723, United States
4 The Solid-State Institute, Technion – Israel Institute of Technology, 32000 Haifa, Israel

Email: *bekenstein@technion.ac.il*

Selected Area Electron Diffraction (SAED):

One of the characterization methods used in order to determine the chemical identity of the synthesis product is SAED. The electron diffraction of the selected area shown in figure S1A presented a poly-crystalline ring pattern. This is expected from selected area of multiple NPs. The assignment of the ring pattern was done by the matching the ratios of one ring radius to other rings in the diffraction pattern. This ratio needs to follow the ratio of d-spacing from different crystallographic plane group of the matched structure. By this method we assigned the orange diffraction rings as shown in figure S1B to $Cs_2AgInCl_6$ NCs. The remaining green rings do not match to the $Cs_2AgInCl_6$ structure and could be assigned to metallic silver of AgCl byproduct. In this work we were interested to learn the identity of the spherical NPs decorating the rectangular shaped $Cs_2AgInCl_6$ NCs. The assignment of the green rings in the electron diffraction to metallic silver NPs matches with the results from other characterization methods, such as energy dispersive X-ray spectroscopy (EDX).

Table S1. Selected area electron diffraction $Cs_2AgInCl_6$ NCs phase assign. The matching of the different phases was done by ratios of the ring pattern radius.

Ring	Material	{hkl}
**1**	$Cs_2AgInCl_6$	220
**2**	$Cs_2AgInCl_6$	222
**3**	$Cs_2AgInCl_6$	400
**4**	AgCl / Ag^0	111
**5**	$Cs_2AgInCl_6$	422
**6**	AgCl / Ag^0	220
**7**	$Cs_2AgInCl_6$	620

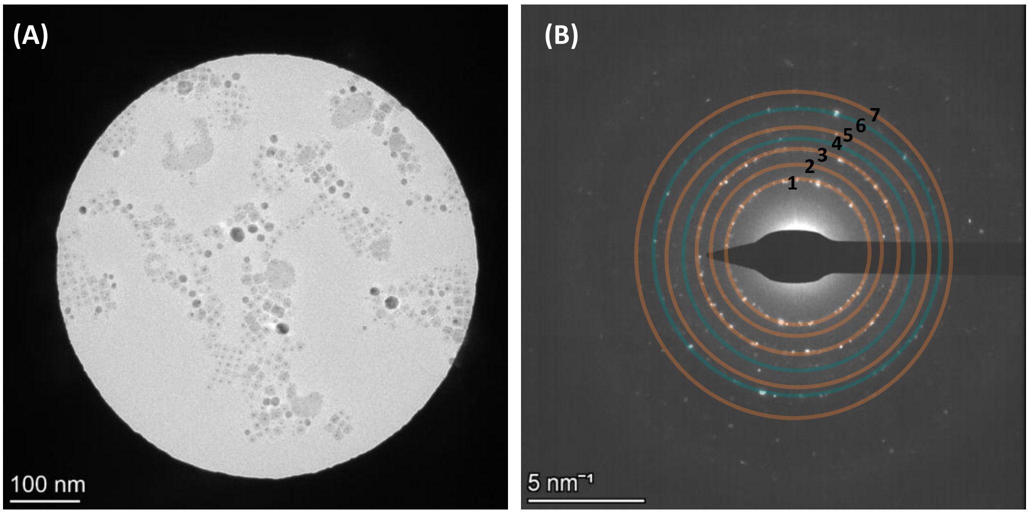

Figure S1. (A) Low magnification TEM micrograph of selected area diffraction of $Cs_2AgInCl_6$ NCs. **(B)** SAED of $Cs_2AgInCl_6$ NCs presenting polycrystalline ring pattern. The SAED reveals two phases assigned to $Cs_2AgInCl_6$ and metallic Ag or AgCl.

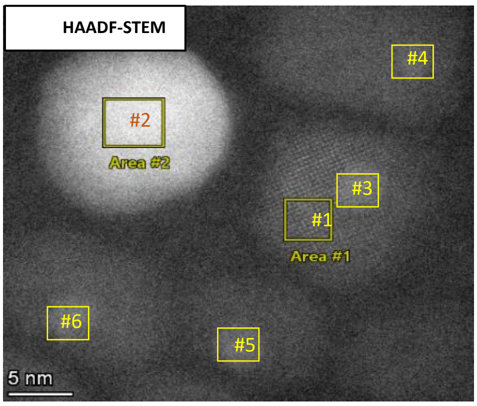

Figure S2. HAADF-STEM micrograph of $Cs_2AgInCl_6$ NCs with selected areas for EDX elemental analysis. The areas include cuboids shaped NP, high contrast spherical NP and high contrast NP decoration.

Table S2. EDX elemental analysis of areas in figure S2. Stichometry confirmed $Cs_2AgInCl_6$ composition for the cuboids shaped NP and metallic silver for the composition of the spherical NPs. The change in the silver NPs could be assigned to the double perovskite composition at the small selected area of the EDX.

Element	Perovskite (area 1) atomic faction [%]	High contrast NP (area 2) atomic fraction [%]	NP decoration (area 3) atomic faction [%]	NP decoration (area 4) atomic fraction [%]	NP decoration (area 5) atomic fraction [%]	NP decoration (area 6) atomic fraction [%]
Cs	20.96±2.87	1.98±0.43	13.68±2.79	19.44±3.07	7.64±1.97	7.80±2.83
Ag	8.42±1.25	92.52±20.16	41.91±7.64	23.70±3.84	55.31±10.88	53.65±11.78
In	10.52±1.53	2.37±0.53	4.09±1.74	5.35±1.30	0.00±1.44	3.14±2.58
Cl	60.10±5.24	3.13±0.64	40.31±6.74	51.51±6.09	37.04±7.22	35.41±9.08

Primary Silver Decorations Morphological analysis:

In order to understand the location of the large primary silver NPs in the $Cs_2AgInCl_6$ NCs, high resolution HAADF-STEM imaging was conducted with different tilts of the TEM grid. HAADF-STEM images of tilted nanocubes (shown in Figure S3) shows that after tilts at high angles of 44° and 50° it can be seen that the projected image of primary silver decoration was outside of the boundaries of the rectangular $Cs_2AgInCl_6$ NCs. This change of the projected images of primary silver decorations is possible if they are located at the center of the surface of the face of the cube. These silver NPs are not located inside the cube as may seemed at first glance when looking at projected images at 0° tilts (perpendicular to the sample). This observation of the location of the primary silver decorations is odd considering their large size and the

effective ligand concentration at the face of the $Cs_2AgInCl_6$ NCs. One possible explanation for such unfavorable location is that the primary silver NPs predate the formation of the $Cs_2AgInCl_6$ NCs.
Additionally, from high resolution TEM of the central Ag decorations we discovered evidence to suggest that they are in fact poly-crystalline. TEM of the central Ag NPs (as shown in figure 3D) revile several regions with different lattice distances. This observation is likely the result of different crystallographic planes for different Ag crystals coagulated together to form the monolithic central silver NP. The reason for such polycrystallinity of the central decorations might be in order to expose favorable facets of the silver crystal, similar to penta-twining seen in Ag nanowires [1,2].

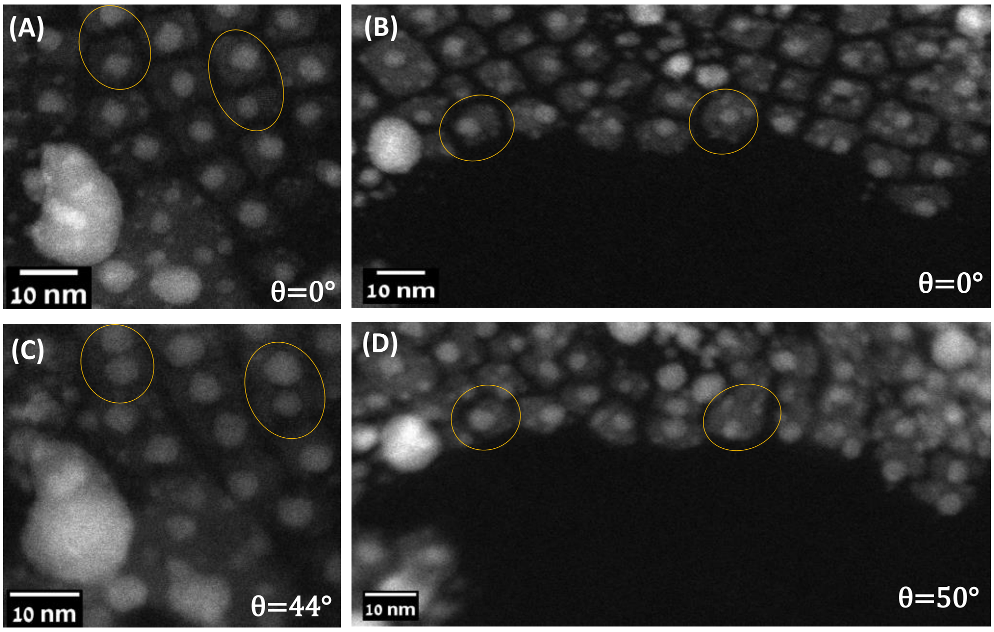

Figure S3. HAADF-STEM micrograph of $Cs_2AgInCl_6$ NCs with silver NPs decorations, **(A-B)** before and **(C-D)** after tilts at high angles of 44° and 50° respectively. After tilts at high angles, it can be seen that the primary silver nanoparticles are partly located outside the perovskite projected image.

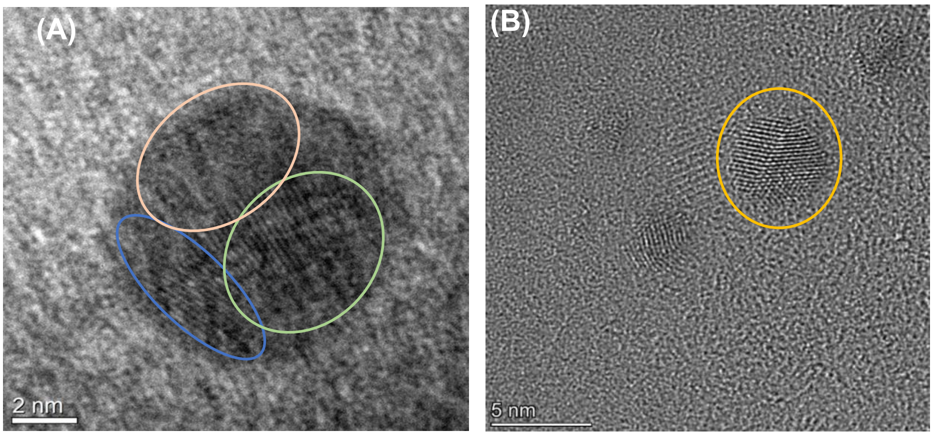

Figure S4. (A-B) High-resolution TEM micrograph of silver NPs decorating $Cs_2AgInCl_6$ nanocube. The silver is revealing a polycrystalline structure of several distinct different crystallographic planes.

<u>Silver NPs formation During Perovskite Synthesis:</u>

Several characterization methods were used in order to determine the part of the $Cs_2AgInCl_6$ NCs synthesis in which the silver NPs form. Sample of the end product of the synthesis was characterize using x-ray spectroscopy (XPS). In the XPS of Ag 3d orbital (shown in figure S5A), two different oxidation states of the Ag are identified. Ag^+ with binding energy of 367.9eV and metallic Ag with binding energy of 368.4eV. This indication of metallic silver prior to TEM and electron beam exposure, is contradictory to the previously hypothesized origin of these silver NPs. Other reaserches[3,4] hypothesized that the silver NPs form by post synthesis electron beam reduction. Therefore, it seems that the silver NPs formation occurs during some stage of the synthesis. Dose dependent analysis of the Ag signal in the XPS shows no correlation between the X-ray exposure time and the peak intensity. The same was done in C signal of the XPS (figure S6). This shows that the silver signal is not a result of the X-ray exposure. Some indications of silver NPs were identified at the complexation stage of the reaction, prior to the injection of chloride to the reaction solution. These indications of the presence of silver NPs (silver plasmon and TEM images) were confirmed by XRD on a sample from the complexation solution after the color change. The diffraction pattern matches with metallic silver. The width of the spectral peaks could be explained by broadening due to the small size of the silver colloids. The broadening of the spectral peaks of the silver NPs could be reduced as background noise in the end product XRD. At the moment of chloride injection, a rapid nucleation of $Cs_2AgInCl_6$ NCs on the preexisting silver NPs occurs with consumption of the silver NPs. This consumption is evident by the mean size of the silver NPs before and after chloride injection (see figure S7C). however, in reaction with short time of reaction time after the injection of chloride, many silver NPs are located outside of the perovskite NCs. This suggest that the consumption of silver NPs is partial. furthermore, likelihood that the original silver NPs population

consumed and then a new population is created through degradation of $Cs_2AgInCl_6$ NCs is low. This contradicts the theory of the origin of silver NPs as part of the DP degradation as described in the silver-bismuth system[5].

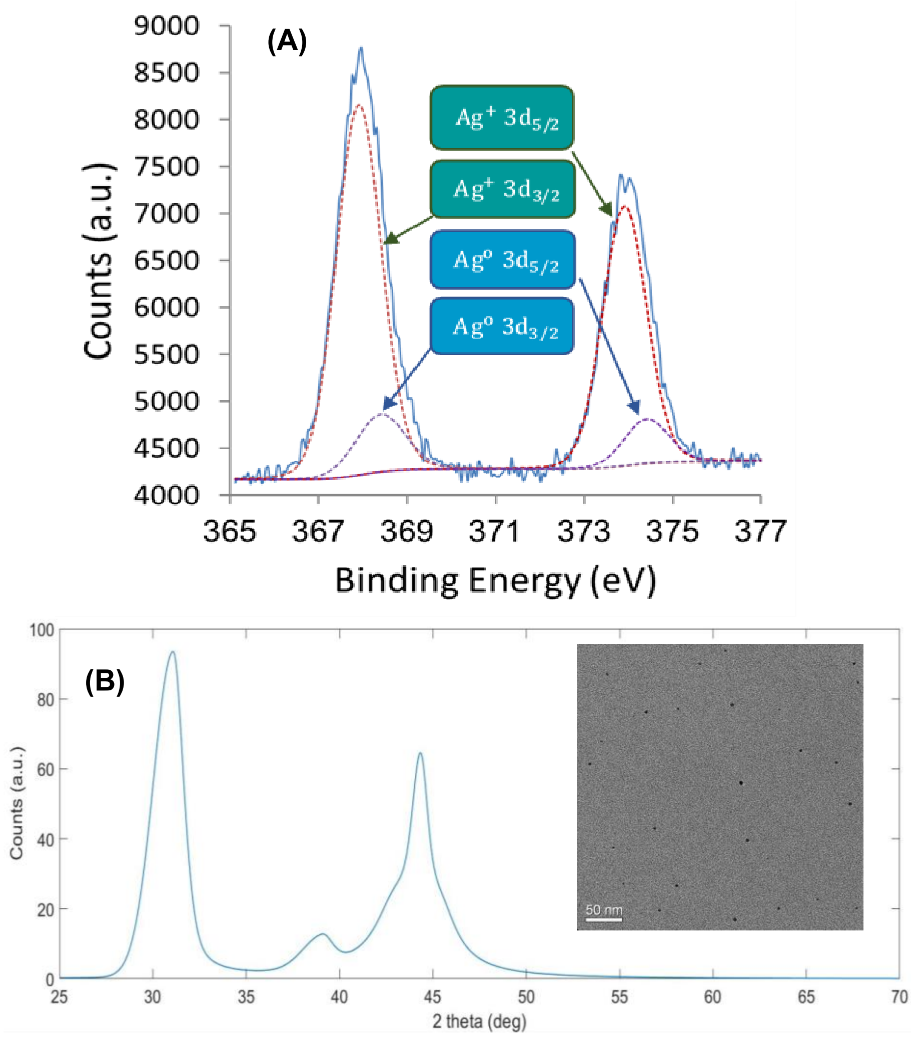

Figure S5. (A) High Resolution XPS spectrum of Ag 3d measured. A spin-orbit splitting of 6eV is measured between the 5/2 and 3/2 peaks. Two different oxidation states of the Ag are identified: Ag^{+} (BE=367.9eV) and metallic Ag (BE=368.4eV). This confirms the reduction of the silver ion precursor into metallic phase prior to any TEM characterization. **(B)** XRD pattern of metallic silver NPs from sample from the reaction solution at the complexation stage prior to the injection of chloride. Inset: Low resolution TEM image of metallic silver NPs formed during the complexation stage.

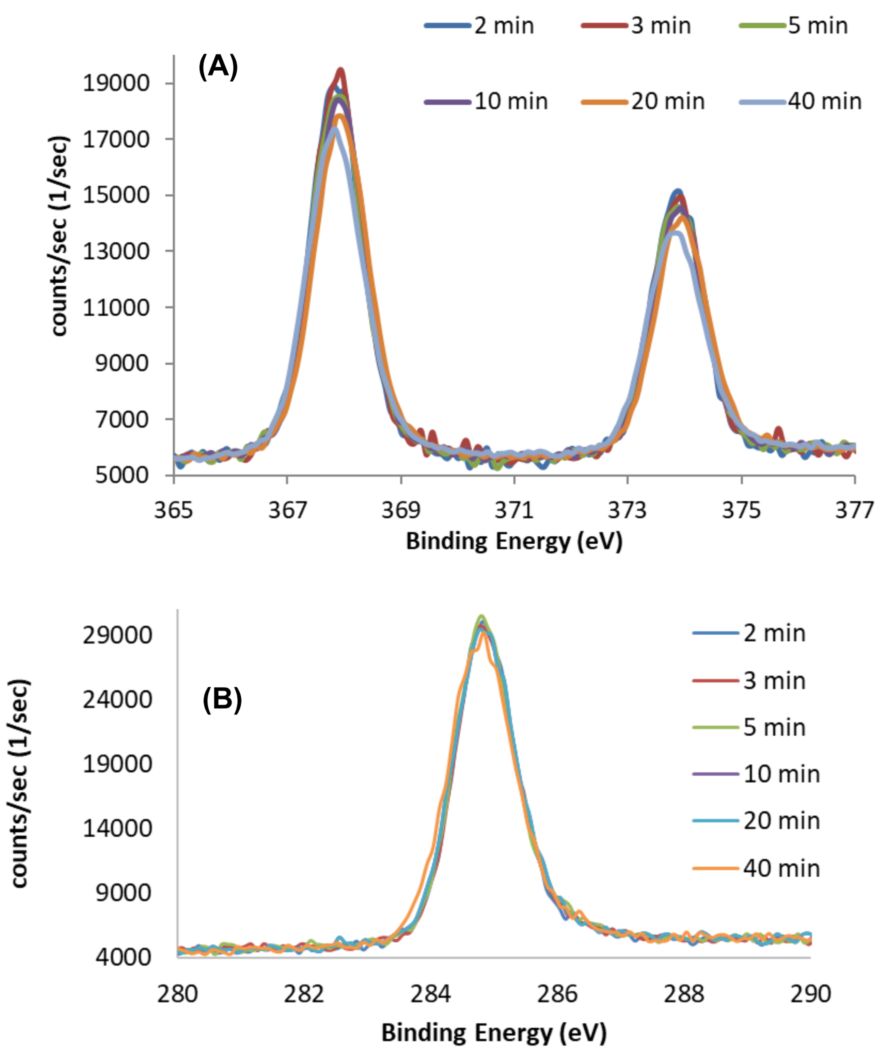

Figure S6. Dose dependent HR XPS spectrums **(A)** Ag 3d orbital measured. A spin-orbit splitting of 6eV is measured between the 5/2 and 3/2 peaks. The intensity of the XPS signal seems independent on the X-ray exposure time. **(B)** C 1s orbital measured for functionality of C-C (BE= 284.2eV). The intensity seems independent on the X-ray exposure time.

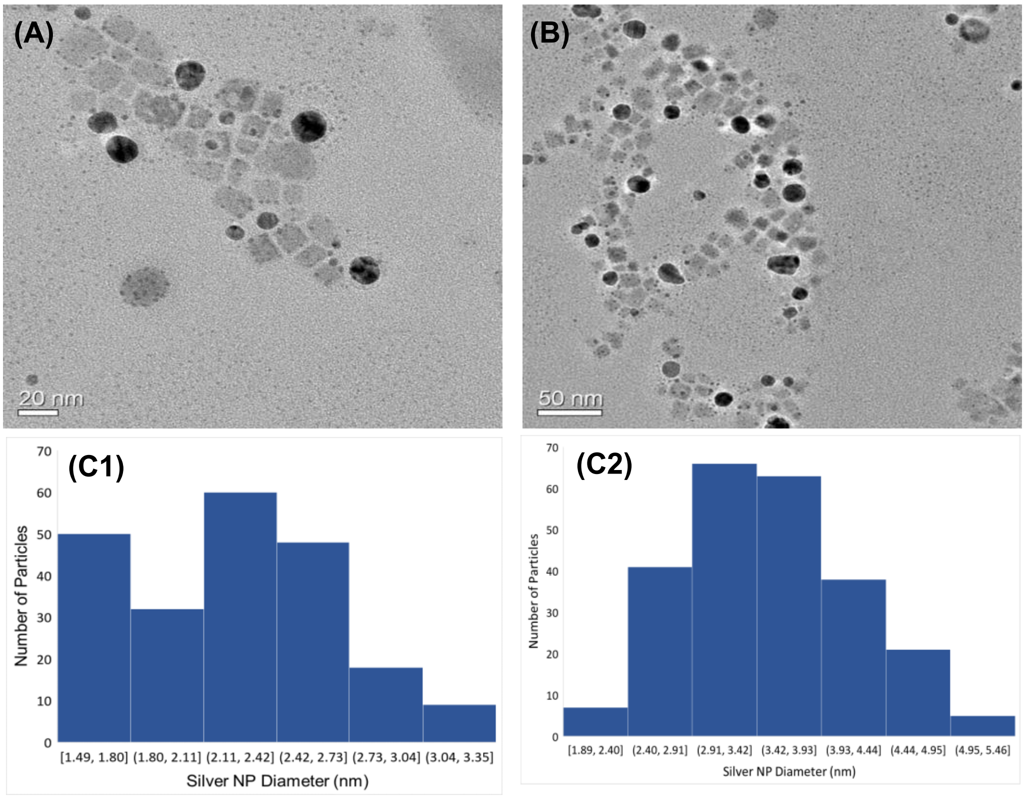

Figure S7. (A-B) Low magnification TEM micrographs of $Cs_2AgInCl_6$ NCs from short reaction time sample. The sample showing many silver NPs outside of $Cs_2AgInCl_6$ NC. **(C1)** Size distribution analysis of Ag NPs from complexation stage (3.52 ± 0.67 nm). **(C2)** Size distribution analysis of Ag NPs decorations shown in fig D1 (2.22 ± 0.44 nm).

$Cs_2AgBiBr_6$, $Cs_2AgBiCl_6$, and $Cs_2AgSbCl_6$ NC DP systems

The main focus of our paper was set on a single model Pb-free DP system of $Cs_2AgInCl_6$. However, in other DP system with silver as the monovalent cation the spherical Ag NPs inhomogeneities have also been observed. In the synthesis of such DP NCs, the color of precursors solution changes color during complexation in a similar way to the plasmon growth in the $Cs_2AgInCl_6$ system (figure S8). The plasmon appears to also red-shift at the moment of benzoyl halide injection by qualitative observation. Unfortunately, the kinetics of these reactions do not allow for a detailed spectroscopy of the shifted plasmon. The complexation stage plasmon was obtained however together with the end product NCs absorption spectrum as seen in figure S9. TEM characterization of these NCs systems (figure S10), shows the commonality of the Ag NPs in these materials as well. This analogy between several Ag based DP NCs colloidal system, emphasizes the importance of understanding the role of Ag NPs in these systems.

Synthesis of $Cs_2AgBiCl_6$ and $Cs_2AgBiBr_6$ Nanocrystals: In a typical synthesis, 25 mg (0.125 mmol) of cesium acetate, 40 mg (0.25 mmol) of silver acetate, and 95 mg (0.25 mmol) of bismuth (III) acetate were placed into a 20 mL glass vial with a magnetic stirring bar. O-xylene (5 mL), oleic acid (1.25mL) and oleylamine (0.375 mL) were added, and the vial was heated to 100°C or 45°C for the desired amount of time. Benzoyl chloride or bromide (0.200 mL, 1.5 mmol) was injected quickly. Then, the vials were taken out of the oil bath and left to cool to room temperature or quenched in water bath. For measurements, the nanocrystal reaction mixture was precipitated by centrifugation at 12,000 rpm for 10 min. The solution was discarded and the resulting pellet was redispersed in hexane.

Synthesis of $Cs_2AgSbCl_6$ Nanocrystals: In a typical synthesis, 25 mg (0.125 mmol) of cesium acetate, 40 mg (0.25 mmol) of silver acetate, and 75 mg (0.25 mmol) of antimony (III) acetate were placed into a 20 mL glass vial with a magnetic stirring bar. O-xylene (5 mL), oleic acid (1.25mL) and oleylamine (0.375 mL) were added, and the vial was heated to 100°C or 45°C for the desired amount of time. Benzoyl chloride (0.200 mL, 1.5 mmol) was injected quickly. Then, the vials were taken out of the oil bath and left to cool to room temperature or quenched in water bath. For measurements, the nanocrystal reaction mixture was precipitated by centrifugation at 12,000 rpm for

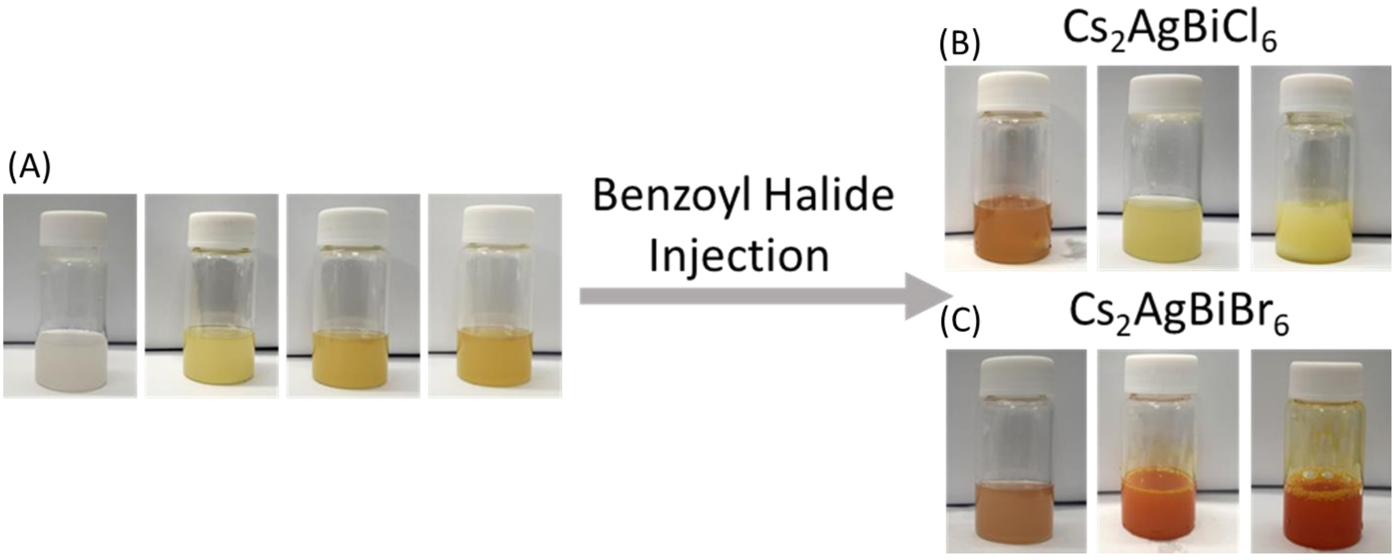

Figure S8. (A) Images of Cs_2AgBiX_6 NCs (X=Cl/Br) precursor solution at different complexation times (before halide injection) at 45 °C, (from left to right) 0min, 5min, 10min, 15min and 20min**. (B)** Images of $Cs_2AgBiCl_6$ NCs reaction solution after chloride injection at different reaction times at 45 °C, (from left to right) 0sec, 1sec, and 3sec. **(C)** Images of $Cs_2AgBiBr_6$ NCs reaction solution after bromide injection at different reaction times at 45 °C, (from left to right) 0sec, 1sec, and 4sec.

10 min. The solution was discarded and the resulting pellet was redispersed in hexane.

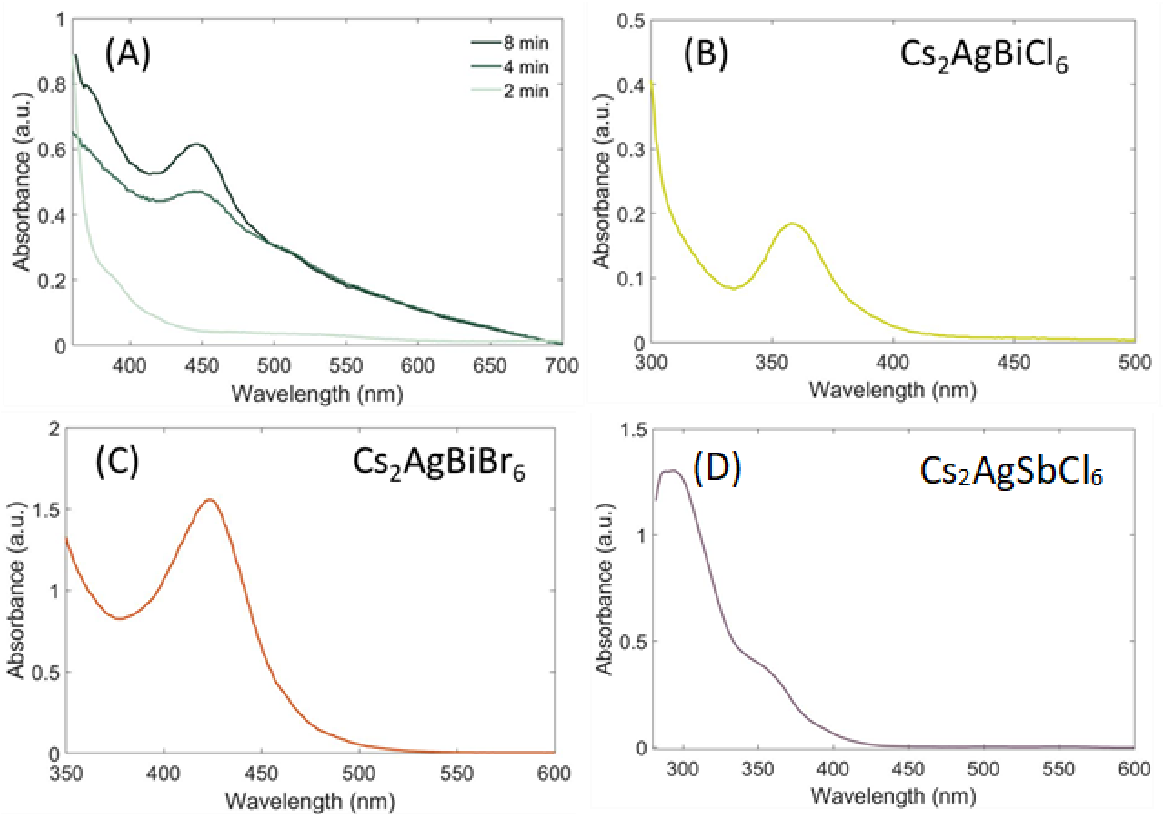

Figure S9. (A) Absorbance spectrum of 45 ^{o}C Cs_2AgBiX_6 NCs (X=Cl/Br) precursors solution at different times at the complexation stage (before halide injection). Formation of metallic silver NPs is indicated by the increase in localized surface plasmon resonance (LSPR) peak. **(B)** $Cs_2AgBiCl_6$ NCs absorbance spectrum. **(C)** $Cs_2AgBiBr_6$ NCs absorbance spectrum. **(D)** $Cs_2AgSbCl_6$ NCs absorbance spectrum.

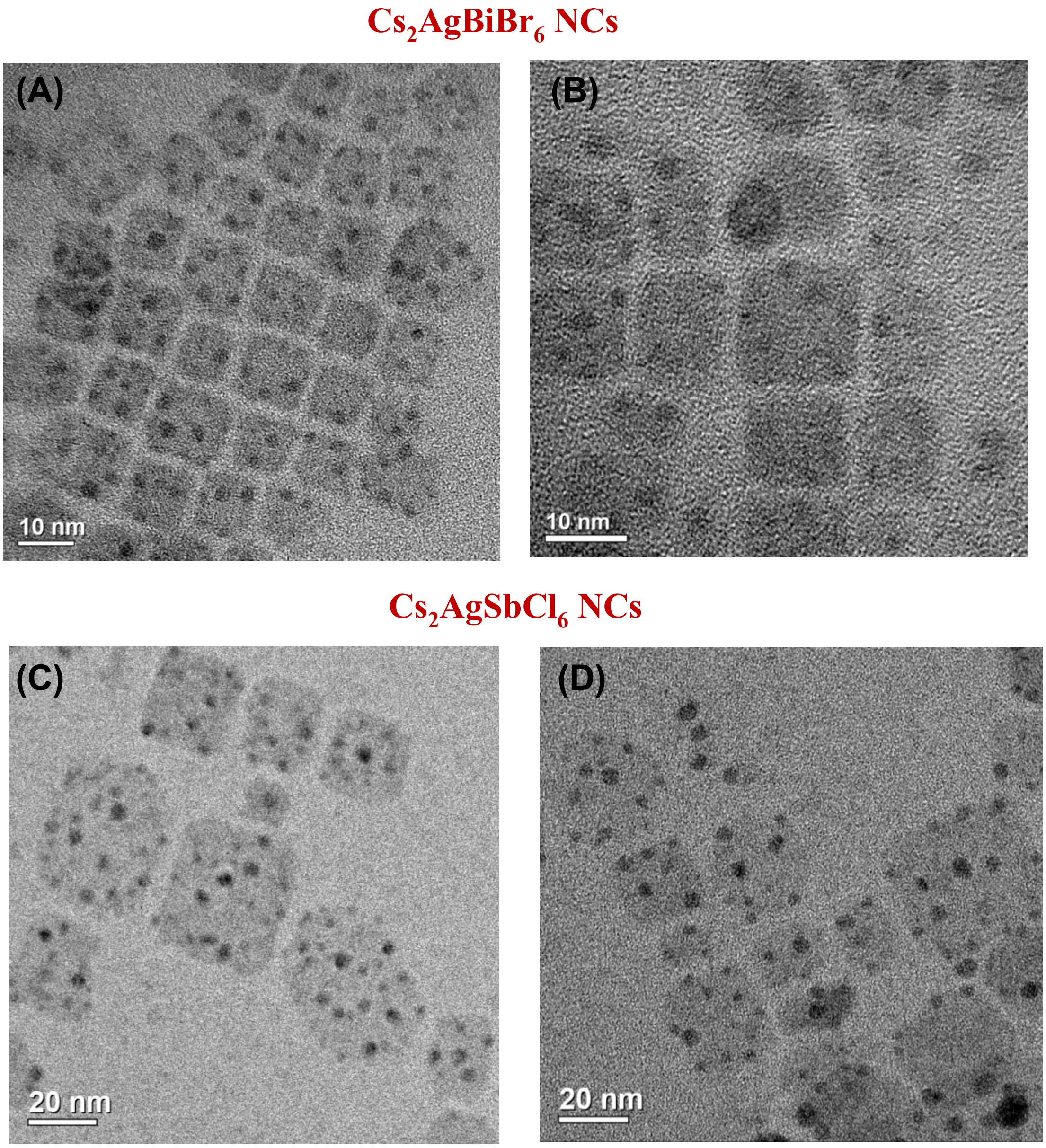

Figure S10. (A-B) TEM micrographs of $Cs_2AgBiBr_6$ NCs with metallic Ag NPs decorations. **(C-D)** TEM micrographs of $Cs_2AgSbCl_6$ NCs with metallic Ag NPs decorations.

References:

1. Zhang, S. H. *et al.* Growth of silver nanowires from solutions: A cyclic penta-twinned-crystal growth mechanism. *J. Phys. Chem. B* (2005).
2. Zhang, J., Langille, M. R. & Mirkin, C. A. Synthesis of silver nanorods by low energy excitation of spherical plasmonic seeds. *Nano Lett.* (2011).
3. Dang, Z. *et al.* In Situ Transmission Electron Microscopy Study of Electron Beam-Induced Transformations in Colloidal Cesium Lead Halide Perovskite Nanocrystals. *ACS Nano* (2017).
4. Locardi, F. *et al.* Colloidal Synthesis of Double Perovskite $Cs_2AgInCl_6$ and Mn-Doped $Cs_2AgInCl_6$ Nanocrystals. *J. Am. Chem. Soc.* (2018).
5. Bekenstein, Y. *et al.* The Making and Breaking of Lead-Free Double Perovskite Nanocrystals of Cesium Silver-Bismuth Halide Compositions. *Nano Lett.* **18**, 3502–3508 (2018).
